# Imitation or Polarity Correspondence? Behavioural and Neurophysiological Evidence for the Confounding Influence of Orthogonal Spatial Compatibility on Measures of Automatic Imitation

**DOI:** 10.3758/s13415-020-00860-y

**Published:** 2021-01-12

**Authors:** Kristína Czekóová, Daniel Joel Shaw, Martin Lamoš, Beáta Špiláková, Miguel Salazar, Milan Brázdil

**Affiliations:** 1 Behavioural and Social Neuroscience, Central European Institute of Technology (CEITEC), Masaryk University, Kamenice 5, 625 00 Brno, Czechia; 2 Institue of Psychology, Czech Academy of Sciences, Brno, Czechia; 3 Department of Psychology, School of Life and Health Sciences, Aston University, Birmingham, UK; 4 Multimodal and Functional Neuroimaging, Central European Institute of Technology (CEITEC), Masaryk University, Brno, Czechia; 5 First Department of Neurology, Faculty of Medicine, Masaryk University and St. Anne’s University Hospital, Brno, Czechia

**Keywords:** Automatic imitation, Orthogonal spatial compatibility, Semantic control, Polarity correspondence

## Abstract

During social interactions, humans tend to imitate one another involuntarily. To investigate the neurocognitive mechanisms driving this tendency, researchers often employ stimulus-response compatibility (SRC) tasks to assess the influence that action observation has on action execution. This is referred to as automatic imitation (AI). The stimuli used frequently in SRC procedures to elicit AI often confound action-related with other nonsocial influences on behaviour; however, in response to the rotated hand-action stimuli employed increasingly, AI partly reflects unspecific up-right/down-left biases in stimulus-response mapping. Despite an emerging awareness of this confounding orthogonal spatial-compatibility effect, psychological and neuroscientific research into social behaviour continues to employ these stimuli to investigate AI. To increase recognition of this methodological issue, the present study measured the systematic influence of orthogonal spatial effects on behavioural and neurophysiological measures of AI acquired with rotated hand-action stimuli in SRC tasks. In Experiment 1, behavioural data from a large sample revealed that complex orthogonal spatial effects exert an influence on AI over and above any topographical similarity between observed and executed actions. Experiment 2 reproduced this finding in a more systematic, within-subject design, and high-density electroencephalography revealed that electrocortical expressions of AI elicited also are modulated by orthogonal spatial compatibility. Finally, source localisations identified a collection of cortical areas sensitive to this spatial confound, including nodes of the multiple-demand and semantic-control networks. These results indicate that AI measured on SRC procedures with the rotated hand stimuli used commonly might reflect neurocognitive mechanisms associated with spatial associations rather than imitative tendencies.

**Supplementary Information:**

The online version contains supplementary material available at 10.3758/s13415-020-00860-y.

## Introduction

Humans have a tendency to imitate one another during social interaction. Although this often occurs involuntarily and outside of conscious awareness, such behaviour appears to serve important social functions; covert imitation among interactants fosters feelings of affiliation and rapport (Chartrand & Lakin, 2013). For this reason, the past decade has seen a surge of research into the cognitive processes and associated brain systems underlying our tendency to imitate others, and its relationship with other aspects of social behaviour and cognition (for reviews see Cracco et al., 2018; Darda & Ramsey, 2019). While this has advanced our understanding of imitative tendencies enormously, it has also revealed that the experimental stimuli employed frequently to investigate such tendencies can produce behaviours *resembling* imitation visually but differing from it fundamentally. Only by isolating imitation from these pseudo-imitative behaviours can we elucidate its neurocognitive underpinnings. So, the present study performed a systematic behavioural and neurophysiological evaluation of the experimental stimuli used commonly.

Imitation refers to the execution of a body movement that is similar *topographically* to one observed previously or concurrently in another person (Cracco & Brass, 2019; Heyes, 2011). Driven by seminal studies (Brass, Bekkering, & Prinz, 2001; Stürmer, Aschersleben, & Prinz, 2000), researchers often employ stimulus-response compatibility (SRC) procedures to investigate imitation experimentally; individuals are asked to execute actions that are the same (compatible) or different (incompatible) to those observed simultaneously in another person. A compatibility effect is demonstrated reliably in such procedures, whereby individuals are quicker and more accurate at executing actions while observing compatible compared with incompatible actions. This is referred to as automatic imitation (AI), and greater AI is taken as an experimental index of an increased tendency to imitate the actions of others involuntary (Heyes, 2011). For this reason, SRC tasks are employed frequently to assess the relationship between imitative tendencies and other aspects of interpersonal behaviour and social functioning (for a recent review, see Cracco et al., 2018).

Importantly, however, the compatibility effect is driven not only by the topographical similarity between the executed and observed movement, referred to herein as *imitative* compatibility, but also their *spatial* compatibility—that is, the degree to which they are performed towards the same spatial location (Aicken, Wilson, Williams, & Mon-Williams, 2007; Bertenthal, Longo, & Kosobud, 2006; Catmur & Heyes, 2011; for a review see Cracco et al., 2018). Until recently, the large majority of SRC procedures required participants to execute index- and middle-finger lifting and/or tapping movements of their right hand positioned horizontally, whilst observing corresponding finger movements of a model’s left hand oriented along the same plane. In this setup, the response and stimulus hand face one another in a mirror-like fashion, with index- and middle-finger movements both executed and observed towards the left and right of the stimulus display, respectively. As such, the compatibility effect might simply reflect our tendency to respond in the direction of a stimulus, rather than to imitate the observed action (Simon, 1969). The strength of this spatial confound is demonstrated when the executed and observed actions are both performed by right hands oriented horizontally, placing imitative and spatial compatibility in direct opposition; this results in a marked reduction, even a partial reversal, of the compatibility effect (Bertenthal et al., 2006; Boyer, Longo, & Bertenthal, 2012). By measuring AI in response to stimuli that confound imitative- with these simple spatial-compatibility effects, studies are likely to confuse imitative tendencies with other nonsocial phenomena (Heyes, 2011; but see Boyer et al., 2012; Catmur & Heyes, 2011).

A growing awareness of the strong confounding effect that simple spatial compatibility exerts on measures of AI in SRC tasks has led several studies to attempt to control for this methodological issue. One approach used increasingly to investigate the neurocognitive mechanisms behind imitative tendencies isolated from any simple spatial-compatibility effects is to rotate a left stimulus hand 90° counter-clockwise from participants’ perspective (Cook & Bird, 2012; de Guzman, Bird, Banissy, & Catmur, 2016; Farwaha & Obhi, 2020; Galang & Obhi, 2020; Gordon et al., 2020; Hogeveen & Obhi, 2013; Hogeveen et al., 2014). In this orthogonal setup, the fingers of the horizontal response hand move up and down but those of the vertical stimulus hand move left and right, which is considered a way of controlling for any nonsocial spatial influences. A new confounding influence is introduced when participants use their right hand to respond to this rotated stimulus hand; however, it is now well established that an up-right/down-left advantage emerges on SRC procedures when a horizontal response set is mapped onto a vertical stimulus display (Cho & Proctor, 2003). This *orthogonal* spatial-compatibility effect is believed to reflect an asymmetric coding of stimulus and response alternatives (Cho & Proctor, 2002, 2005). When presented vertically, upward stimuli are coded with greater salience, or positive polarity, compared with downward stimuli, the latter of which assume relatively negative polarity. Likewise, when horizontal responses are made with a right hand, rightward responses are coded with positive polarity and leftward responses with negative polarity. Through polarity correspondence, or salience matching, an up-right/down-left advantage will emerge when mapping horizontal responses to vertical stimuli. Because studies employing SRC procedures to measure AI in finger movements have relied almost exclusively on right-handed participants, AI will be confounded by this nonsocial polarity-correspondence effect; the observation of index- and middle-finger movements performed by a left stimulus hand oriented counterclockwise (downwards and upwards) will facilitate quicker execution of index- and middle-finger movements not just because of their topographical similarity, but also because of their corresponding spatial positioning (leftward and rightward; e.g., Proctor & Xiong, 2015). Indeed, an up-right/down-left advantage has been shown in response to a range of nonsocial stimuli, such as location words (“right”/“left”; “above”/“below”), high- and low-pitch tones, and directional arrows (Proctor & Cho, 2006; Proctor & Vu, 2006; Proctor & Xiong, 2015).

In a previous behavioural study with a large, right-handed sample, we investigated the extent to which orthogonal spatial-compatibility effects might confound AI measured with SRC procedures that employ rotated hand stimuli (Shaw, Czekóová, & Porubanová, 2017). This revealed that AI was reduced by nearly 40% in response to a right compared with a left stimulus hand rotated 90° counter-clockwise, the former of which places the influences of imitative- and orthogonal spatial-compatibility effects in opposition. Interestingly, this pattern was partially (nonsignificantly) reversed for clockwise-rotated hand stimuli, whereby an up-right/down-left advantage would confound any imitative effects for a right stimulus hand but exert an opposing influence for a left-hand stimulus. These earlier results converge with recent meta-analytic data to demonstrate the powerful influence that orthogonal spatial-compatibility effects can exert on measures of AI acquired in SRC procedures (Cracco et al., 2018) and their potential to obscure relationships between imitation and other aspects of social cognition (Shaw et al., 2017). This raises questions over the associations reported in studies using rotated hand stimuli between AI and social behaviour, as acknowledged in more recent research (Galang & Obhi, 2020; Gordon et al., 2020).

While some behavioural studies suggest that imitative- and simple spatial-compatibility effects reflect distinct cognitive mechanisms (Bertenthal & Scheutz, 2013; Cooper, Catmur, & Heyes, 2013), others indicate that they emerge from a common process (Catmur & Heyes, 2011; Cooper et al., 2013). The same contention applies to neuroscientific findings that have dissociated between these effects; during SRC tasks, the medial and lateral prefrontal cortex (PFC), inferior frontal gyrus (IFG), inferior parietal lobule, and temporo-parietal junction have all been implicated in imitative tendencies specifically (Mengotti, Corradi-Dell’Acqua, & Rumiati, 2012; Mengotti, Ticini, Waszak, Schütz-Bosbach, & Rumiati, 2013; Sowden & Catmur, 2015), whereas other studies report that simple spatial-compatibility effects engage a fronto-parietal network encompassing many of the same brain regions (e.g., IFG; Darda, Butler, & Ramsey, 2019; Marsh, Bird, & Catmur, 2016). In an attempt to reconcile these discrepancies, a recent meta-analysis was performed on neuroimaging studies employing stimuli that dissociate among these sources of AI (Darda & Ramsey, 2019). This confirmed that simple spatial compatibility preferentially engages constituent nodes of the multiple-demand network—a fronto-parietal collection of brain regions that respond to a wide range of cognitively demanding tasks, including spatial interference tasks (Cieslik, Mueller, Eickhoff, Langner, & Eickhoff, 2015; Duncan, 2010; Fedorenko, Duncan, & Kanwisher, 2013). In this light, neuroscientific investigations employing SRC procedures with rotated stimuli that confound imitative- with orthogonal spatial-compatibility effects (de Guzman et al., 2016; Santiesteban, Banissy, Catmur, & Bird, 2012, 2015) may have elicited brain responses associated with cognitive processes unrelated to, or at least unspecific to, imitative tendencies. To the best of our knowledge, however, no study has investigated the neural responses associated specifically with *orthogonal* spatial-compatibility effects afforded by rotated hand stimuli, despite meta-analytic evidence of their strong influence on measures of AI (Cracco et al., 2018).

Although some recent studies have attempted to control for simple spatial-compatibility effects (Darda, Butler, & Ramsey, 2018, 2020; Marsh et al., 2016; Trilla, Wnendt, & Dziobek, 2020), very few implement measures to avoid the confounding influence of non-social orthogonal spatial compatibility (Gowen, Bolton, & Poliakoff, 2016). We suspect that this is due the lack of systematic investigations into this confounding effect and apparent inconsistencies in the few available; other than our own (Shaw et al., 2017), only Jiménez et al. (2012) have explored the influence of orthogonal-compatibility effects on AI measured in SCR procedures. This earlier investigation reported no confounding influence in response to clockwise-rotated left and right stimulus hands, but this might be due to their underpowered sample (*N* = 17). Moreover, no study has investigated the brain processes associated specifically with orthogonal-compatibility effects on SRC tasks. To address this sparsity, the present study first assessed the reproducibility of the orthogonal-compatibility effects revealed in our earlier work by revisiting behavioural data collected more recently from an even larger independent sample of right-handed individuals (for details, see Shaw et al., 2020). In this study, the same SRC procedure was used as a measure of AI to assess associations between imitative tendencies and other facets of social cognition. Consistent with our earlier results (Shaw et al., 2017), spatial orientation was manipulated in a between-subject manner while hand anatomy was a within-subject factor. We predicted that AI would be significantly greater in response to a left compared with a right stimulus hand rotated counterclockwise, but marginally greater for right relative to a left clockwise-rotated hand. To assess the influence of orthogonal spatial-compatibility effects more systematically, and investigate their neurophysiological correlates, in a second experiment we re-recruited a subsample of these right handers and compared behavioural and electrocortical indices of AI in response to left and right stimulus hands rotated both counterclockwise and clockwise as a within-subject manipulation. Given the meta-analytic results of Darda and Ramsey (2019), we hypothesised that nodes of the multiple-demand brain network associated with domain-general cognitive processes would exhibit differential responses to hand stimuli according to their affordance of orthogonal spatial compatibility.

## Experiment 1

### Method

#### Participants

The sample consisted of 300 right-handed participants (120 males; M_age_ = 23.1 [SD = 3.2] years). None of the participants reported neurological or psychiatric diagnosis, and all had normal or corrected-to-normal vision. The sample was recruited from students and associates of Masaryk University, Czech Republic. The study was approved by the Ethical Review Board of the Institute of Psychology, Academy of Sciences of the Czech Republic. Written, informed consent was acquired from each volunteer before their participation in the study. Participants received 300 Kč (approx. €12) upon completion of the experiment.

#### Materials

**Fig. 1:**
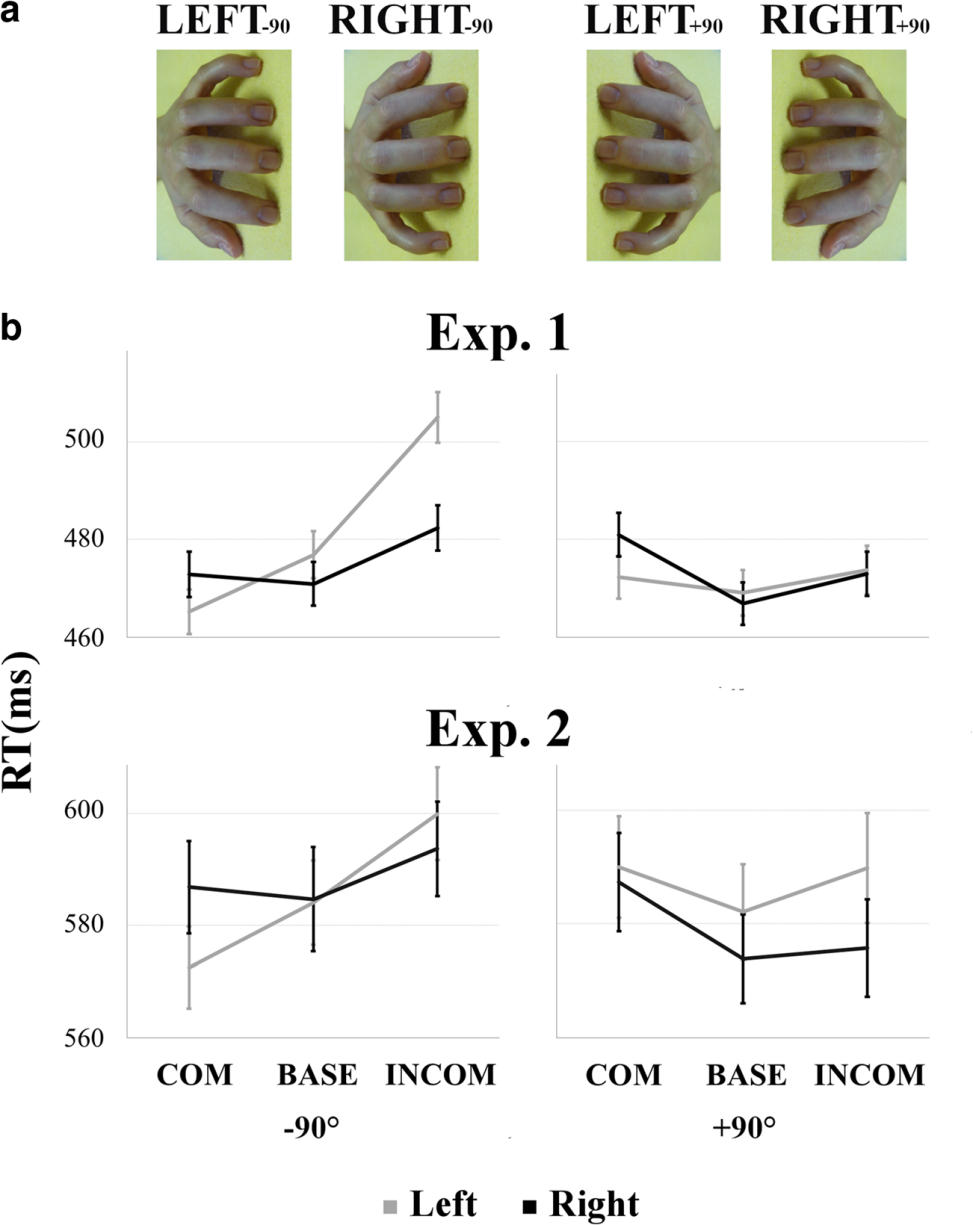
**a** Stimulus hands employed in the SRC task. Left and right stimulus hands were presented in counter-clockwise and clockwise rotations. **b** For Experiments 1 and 2, respectively, response times are presented for Compatible (COM), Baseline (BASE), and Incompatible (INCOM) trials in response to left (*grey*) and right (*black*) stimulus hands presented at a counterclockwise (−90°; *left*) and clockwise orientation (+90°; *right*)

A stimulus-response compatibility (SRC) task was administered as the first task in a larger battery of performance-based and self-report measures of social cognition under laboratory conditions, full details of which are provided in Shaw et al. (2020). On this SRC task, participants were required to lift the index or middle finger of their right hand as quickly as possible in response to a coloured dot (imperative stimulus) while observing task irrelevant finger movements performed simultaneously by a stimulus hand. All trials began with the stimulus hand resting on a flat surface, signalling that participants should depress both the left and right directional arrows on a standard keyboard with their right index and middle finger, respectively. After a variable interval of 800, 1,600, or 2,400 ms, selected randomly, this warning stimulus was replaced with a static image of the same hand after performing either an index or middle finger extension. In this end-point image, a green or red dot was presented between the index and middle finger of the stimulus hand, the colour of which served as the imperative stimulus; it signalled whether the participant should extend their own index or middle finger, thereby releasing the corresponding key (e.g., a green dot required an index finger response, while a red dot indicated a middle finger response; the colour-finger pairing was counterbalanced across participants). A blank screen was presented as soon as a movement was detected, with accuracy and response time (RT) both recorded (Supplementary Figure S1). Combinations of the imperative stimulus and observed finger movement performed by the stimulus hand resulted in 30 compatible (COM; the same finger movement was both signalled and observed), 30 incompatible (INCOM; opposite finger movements were signalled and observed), 20 baseline (BASE; a movement was signalled, but not observed), and 10 catch trials (no imperative stimulus was presented and so no movement was signalled, but the stimulus hand performed one of the two finger actions). We focus only on COM, INCOM, and BASE trials herein. Two blocks of 90 randomised trials were presented. The observed finger actions were performed by a model’s left or right hand rotated 90° counter-clockwise (LEFT_-90_ and RIGHT_-90_) or clockwise (LEFT_+90_ and RIGHT_+90_) from the participant’s perspective (Figure 1a). Each block presented either a left or right stimulus hand, the order of which was counterbalanced across participants. In this mixed design, the left and right stimulus hands were rotated counterclockwise for approximately half the sample (Group_-90_; *n* = 142) and clockwise for the remaining participants (Group_+90_; *n* = 152).

### Results

For each individual, we considered RTs only for correct responses within 3 standard deviations (SD) of their overall mean. Seven participants with an overall mean RT beyond three SDs of the sample mean in either block were omitted from subsequent analyses. The final sample thus comprised 293 participants (119 males).

Separate mixed ANOVAs were performed on RTs and accuracy data, with the within-subject factors *Condition* (COM, BASE, INCOM) and *Stimulus Hand* (LEFT, RIGHT), and the between-subject factor *Orientation* (−90°, +90°). Results of the analysis applied to RT data are presented below, but those for accuracy are presented in Supplementary Materials for the sake of brevity (Supplementary Figure S2). Analyses were performed with SPSS 24. Where significant main effects or interactions emerged, follow-up pairwise comparisons were performed with Bonferroni correction. Greenhouse-Geisser corrections are reported wherever the assumption of variance homogeneity was violated. To aid interpretation, we refer to differences among conditions according to the effects they represent: First and foremost, longer RTs on INCOM compared with COM trials express a compatibility effect, or automatic imitation (AI). Second, longer RTs on INCOM compared with BASE trials reflect the inhibitory effect of observing an incompatible action. Third, shorter RTs on COM relative to BASE trials indicate a facilitatory effect of observing an action that is compatible with the response signalled by the imperative stimulus. Where these differences appeared in the opposite direction (e.g., INCOM_RT_ < COM_RT_,), we refer to these effects being *reversed*. Given our predictions, we were interested primarily in the three-way *Condition*-by-*Stimulus Hand*-by-*Orientation* interaction; specifically, we expected AI, as indexed by a positive compatibility effect, to be significantly greater for the LEFT than the RIGHT stimulus hand presented counterclockwise (Group-_90_) but slightly larger for the RIGHT compared with the LEFT clockwise-rotated stimuli hand (Group-_90_).

#### Response time

When averaging across all trial types, there was no significant difference in RTs between the groups exposed to the counterclockwise (481.01 [±4.48] ms) or clockwise stimulus rotation (474.87 [±4.06] ms; *t*[291] = 1.02; *p* = 0.309; *d* = 0.12).

There was no significant main effect of *Stimulus Hand* (F[1, 291] = 2.23, *p* = 0.136, *r* = 0.09) or *Orientation* (F[1, 291] = 1.08, *p* = 0.300, *r* = 0.06), demonstrating that RTs did not differ statistically between left and right stimulus hands or counterclockwise and clockwise rotations. There was, however, a significant main effect of *Condition* [F[2, 582] = 53.39, *p* < 0.001]. When collapsing across both stimulus hands and rotations, follow-up contrasts revealed a strong compatibility (F[1, 291] = 63.94, *p* < 0.001, *r* = 0.42) and inhibitory effect (F[1, 291] = 90.67, *p* < 0.001, *r* = 0.49) but no facilitation (F[1, 291] = 2.21, *p* = 0.138, *r* = 0.09).

A significant *Condition*-by-*Stimulus Hand* interaction also was observed (F[1.85, 539.01] = 30.27, *p* < 0.001), with contrasts showing the following: a compatibility effect was expressed for the left (20.59 ms, *p* < 0.001) but not the right stimulus hands (0.78 ms, *p* > 0.999; F[1, 291] = 46.97, *p* < 0.001, *r* = 0.37), a larger inhibitory effect was observed in response to the left (16.40 ms, *p* < 0.001) relative to the right stimulus hands (8.79 ms, *p* < 0.001; F[1, 291] = 9.25, *p* = 0.003, *r* = 0.18), and the facilitation effect expressed in response to the left stimulus hands (−4.20 ms, *p* = 0.05) was *reversed* for the right stimulus hands (8.01 ms, *p* < 0.001; F[1, 291] = 28.74, *p* < 0.001, *r* = 0.30).

There was a small but significant *Stimulus Hand*-by-*Orientation* interaction (F[1, 291] = 6.91, *p* = 0.009, *r* = 0.15), but no follow-up contrasts reached statistical significance (*p* ≥ 0.094). In contrast, a strong *Condition*-by-*Orientation* interaction (F[2, 582] = 56.60, *p* < 0.001) revealed a significant compatibility effect in response to the counter-clockwise (24.66 ms, *p* < 0.001) but not the clockwise rotation (−3.29 ms, *p* = 0.236; F [1, 291] = 109.30, *p* < 0.001, *r* = 0.52). Correspondingly, the inhibitory effect was significantly greater in response to the counterclockwise (19.83 ms, *p* < 0.001) compared with the clockwise orientation (5.36 ms, *p* = 0.012; F[1, 291] = 29.94, *p* < 0.001, *r* = 0.31). Lastly, while facilitation was present for the counterclockwise stimuli rotation (−4.83 ms, *p* = 0.027), a significant *reversal* of this effect was identified for the clockwise rotation (8.64 ms, *p* < 0.001; F[1, 291] = 27.65, *p* < 0.001, *r* = 0.29).

Finally, there was a significant *Condition*-by-*Stimulus Hand*-by-*Orientation* interaction (F[2, 582] = 9.74, *p* < 0.001), with a differential compatibility effect exhibited between stimulus hands and orientations (F[1, 291] = 13.04, *p* < 0.001, *r* = 0.21); specifically, while this effect was present for both stimulus hands in the counter-clockwise orientation, it was significantly greater for the LEFT_-90_ (39.79 ms, *p* < 0.001) than the RIGHT_-90_ stimulus (9.54 ms, *p* = 0.002). Moreover, there was no compatibility effect for the LEFT_+90_ (1.40 ms, *p* > 0.999) and its *reversal* was identified for the RIGHT_+90_ stimulus (−7.97 ms, *p* = 0.008). The inhibitory effect followed a similar pattern; it was greater in response to the LEFT_-90_ (28.16 ms, *p* < 0.001) compared with the RIGHT_-90_ stimulus (11.50 ms, *p* < 0.001; F[1, 291] = 13.10, *p* < 0.001), much smaller for RIGHT_+90_ (6.08 ms, *p* = 0.036) and nonsignificant for LEFT_+90_ (4.63 ms, *p* = 0.248). In contrast, no differences emerged in the facilitation effect between *Stimulus hands* or *Orientations* (F[1, 291] = 0.37, *p* = 0.545, *r* = 0.04). Pairwise comparisons showed that these significant contrasts between stimulus hands were driven solely by differences in RT on COM and INCOM trials; responses to BASE trials did not differ significantly (*p* > 0.05; Figure 1b).

The three-way interaction provides partial support for our hypothesis; it reveals that AI, as indexed by a positive compatibility effect, was significantly stronger in response to the LEFT_-90_ compared with the RIGHT_-90_ stimulus hand. Since topographically similar finger movements performed by the LEFT_-90_ stimulus hand are both imitatively *and* orthogonally compatible, while those of the RIGHT_-90_ stimulus are only imitatively compatible, this suggests that orthogonal spatial influences may indeed contribute to AI elicited by the former stimulus. Surprisingly, however, AI was reversed when responding to the RIGHT_+90_ stimulus, indicating the presence of an alternative influence on behaviour capable of overturning any imitative-compatibility effects.

#### Inverted Efficiency

To investigate whether differences in AI among stimulus hands and rotations were present after taking into account any potential speed-accuracy trade off, we computed inverted efficiency scores (IE; RT/proportion correct) in each condition and then calculated a compatibility effect (INCOM_IE_-COM_IE_). These data were then entered into a 2 (*Stimulus Hand*: LEFT, RIGHT) x 2 (*Orientation*: −90°, +90°) mixed ANOVA. This revealed a significant main effect of both *Stimulus Hand* (F[1, 291] = 40.86, *p* < 0.001, *r* = 0.35) and *Orientation* (F[1, 291] = 98.31, *p* < 0.001, *r* = 0.50); there was a greater compatibility effect when responding to the left (32.37 ms) compared with the right stimulus hands (−2.02 ms), and for the counter-clockwise (37.00 ms) relative to the clockwise orientation (−6.64 ms). More importantly, a *Stimulus Hand*-by-*Orientation* interaction also was observed (F[1, 291] = 5.87, *p* = 0.016, *r* = 0.14); consistent with our predictions, IE exhibited a significantly greater compatibility effect in response to the LEFT_-90_ (60.71 ms) compared with the RIGHT_-90_ stimulus (13.28 ms), suggesting that orthogonal and imitative compatibility combined for the former. The compatibility effect was again *reversed* between LEFT_+90_ (4.04 ms) and RIGHT_+90_ (−17.32 ms), and a one-sample *t*-test confirmed that this negative effect was significantly different from zero (*t*[150] = 3.34, *p* = 0.001, *d* = 0.54, 95% CI [−27.58, −7.06]; Figure 3a). Together with RT and accuracy data, which show reversed facilitation for clockwise-rotated stimulus hands, this confirms that some force other than an up-right/down-left advantage operates with sufficient strength to make topographically similar (compatible) actions performed by the RIGHT_+90_ stimulus interfere with action execution.

### Discussion

The results of Experiment 1 reproduced our previous findings that a left stimulus hand rotated counterclockwise elicits significantly greater AI than a right stimulus hand at the same rotation (Shaw et al., 2017). Because the former (anatomically incompatible) stimulus affords both imitative- and orthogonal spatial-compatibility effects, while the latter places these two sources in opposition, this provides further evidence that AI measured on this task is driven partly by nonsocial, orthogonal spatial influences. This aligns with the findings of other studies that have attempted to dissociate between imitative and confounding spatial influences (Bertenthal et al., 2006; Catmur & Heyes, 2011; Gowen, Bolton, & Poliakoff, 2016; Longo & Bertenthal, 2009). An unexpected pattern emerged for clockwise-rotated stimuli; however, while small but positive AI was elicited by the left hand, it was significantly *reversed* in response to the right stimulus hand. In other words, the observation of compatible actions performed by clockwise-rotated hands, which should facilitate action execution through imitative processes, actually served to *interfere* with performance. These findings provide strong evidence that AI measured in response to the rotated hand stimuli used increasingly in SRC tasks can be driven by influences independent of the topographical or anatomical compatibility between observed and executed movements. This questions the suitability of such stimuli for investigations into (the control of) imitative tendencies.

The reversal in AI, whereby the observation of index- and middle-finger movements performed by a right hand rotated clockwise facilitated the execution of middle- and index-finger actions, rather than topographically equivalent movements, might reflect the influence of *complex* orthogonal spatial compatibility effects. This refers to the well-documented effect in which an up-right/down-left bias on SRC tasks switches to an up-left/down-right bias following a change in the relative positioning of the response and stimulus set (Cho & Proctor, 2002, 2003, 2004). In our experiment, the positioning of the response hand was kept constant, but the observed finger movements were performed leftward of the imperative stimulus (left hemispace) for the counterclockwise rotation or rightwards (right hemispace) for the clockwise rotation. Such complex orthogonal spatial compatibility effects might therefore go some way towards an explanation of the reversal of AI that we observed for the clockwise-rotated stimuli. The multiple asymmetric codes theory (Proctor & Cho, 2006) attempts to explain this effect by proposing that rightward responses are coded with positive polarity (more salience) when made to a vertical stimulus set positioned in left hemispace, but leftward responses become coded with positive polarity if the same stimulus is positioned within right hemispace. Through polarity correspondence, then, rightward responses (middle-finger actions) to our counterclockwise stimuli should be facilitated by the observation of upward stimuli: that is, middle-finger movements of a left stimulus hand and index-finger movements of a right stimulus hand. In contrast, leftward responses (index-finger actions) to the clockwise-rotated hand stimuli should be facilitated by the observation of upward stimuli: index-finger movements performed by a left stimulus hand, and middle-finger movements of a right stimulus hand. Importantly, however, neither complex orthogonal compatibility nor the asymmetric codes theory can account for responses to the right hand in counter-clockwise rotation, which elicited a partial reduction rather than reversal of AI.

The pattern of change that we have observed in both RT and accuracy indices of AI were driven by differences on compatible and incompatible trials; responses on baseline trials remained stable across stimuli. First, a right hand at both rotations and a clockwise rotation of both left and right hands increased RT and decreased accuracy on compatible relative to baseline trials. In other words, observing a topographically similar action performed by these stimulus hands served to inhibit rather than facilitate its execution. Second, RTs were shorter and accuracy improved on incompatible relative to baseline trials for a right stimulus hand rotated counterclockwise; when imitative- and orthogonal spatial-compatibility effects were placed in opposition, the observation of incompatible actions facilitated rather than inhibited their execution. The presence of both facilitatory and inhibitory effects for the left stimulus hand rotated counterclockwise, and an absence of facilitation for a right hand at the same orientation, supports the proposition that spatial influences elicit both processes while imitative compatibility comprises inhibition predominantly (Boyer et al., 2012; Jiménez et al., 2012). Conversely, this framework cannot explain the reversal of facilitation for a right hand rotated clockwise, nor the absence of either process for a left stimulus hand at this orientation (Bertenthal et al., 2006). Instead, our data suggest that both facilitation and inhibition are amenable to orthogonal spatial influences, resulting in differential AI for each stimulus hand. The strong influence of this orthogonal spatial effect, which appears capable of overriding any imitative effects, questions the extent to which AI measured on SRC tasks employing rotated hand stimuli indexes our tendency to imitate others during social interaction.

By demonstrating that AI can be reversed completely, the findings of this first experiment conflict with the view that AI remains present, albeit to a lesser extent, even when spatial influences are controlled (Bertenthal et al., 2006; Catmur & Heyes, 2011; Cracco & Brass, 2019). A closer inspection of the data reported by Darda et al. (2018) and Marsh et al. (2016) shows that AI also can be reversed by simple spatial influences; in both studies, positive AI measured in response to a left stimulus hand positioned horizontally was reversed to negative AI for a right stimulus hand, the latter of which places imitative- and simple spatial-compatibility in opposition. Before discussing this pattern further, however, it is important to acknowledge that the reversal of AI demonstrated in the present study was not observed in our earlier work (Shaw et al., 2017). Because the only difference between the two SRC procedures was the inclusion of a baseline condition, we suggest that strong interindividual variability might be behind these discrepant findings. Our previous investigation identified subgroups defined by their differential sensitivity to imitative- and orthogonal spatial-compatibility effects (Shaw et al., 2017; see also Jiménez et al., 2012). Because orientation of the stimulus hands was manipulated in a between-subject manner in both the previous and current experiments, individual differences may have masked any reliable reversal of AI. To minimise this potential influence, in the subsequent experiment we explored the reproducibility of this reversal by administering the same SRC task comprising all stimulus combinations in an entirely within-subject design.

## Experiment 2

In addition to assessing whether or not the pattern of AI observed in Experiment 1 can be reproduced when stimulus orientation is manipulated in a within-subject manner, providing a more systematic assessment of orthogonal spatial-compatibility effects, the second experiment employed electroencephalography (EEG) to investigate whether these effects also exert an influence on neurophysiological indices of AI. To permit comparisons with the handful of previous studies that have employed EEG to investigate the neural correlates of AI measured on the SCR task, we focus specifically on two stimulus-locked components of the event-related potential (ERP): the N190—a negative wave peaking at approximately 200 ms at lateral occipito-temporal regions, which has been localised within inferior temporal cortex (Thierry et al., 2006), and the P3—a positive wave peaking at 300-500 ms that occurs maximally over parietal regions (Deschrijver, Wiersema, & Brass, 2017b; Nishimura, Ikeda, & Higuchi, 2018; Rauchbauer, Pfabigan, & Lamm, 2018). A greater N190 in response to incompatible relative to compatible trials on the SRC task has been proposed to index low-level visual self-other distinction processes, while a larger P3 amplitude elicited by compatible compared with incompatible actions has been interpreted to reflect the cognitive processes recruited when resolving conflicts between internally and externally generated motor plans (Deschrijver et al., 2017b; Deschrijver, Wiersema, & Brass, 2017a; Rauchbauer et al., 2018).

To identify brain responses sensitive to orthogonal spatial compatibility, we performed high-density EEG to capture electrocortical signals representing early (bottom-up) and late (top-down) cognitive processing that differentiate between pairs of stimulus hands according to their affordance of this spatial confound. Permitting accurate source estimation, this also allowed us to localise the brain regions expressing such sensitivity to orthogonal compatibility. Driven by the results of Experiment 1, we predicted that electrocortical signals sensitive to orthogonal spatial compatibility would differentiate between pairs of stimuli after controlling for anatomy and orientation: ERPs reflecting a compatibility effect (INCOM>COM or COM>INCOM) would be greater for a left stimulus hand rotated counterclockwise compared with both a right hand at the same orientation and the same left hand oriented clockwise; and reversed for a clockwise-rotated right stimulus hand relative to a left hand at the same orientation and the same right hand rotated counterclockwise.

### Method

#### Participants

A sample of 69 right-handed individuals (27 males; M_age_ = 24.1 years, SD = 3.3) was re-recruited from the aforementioned study one year later. This sample size was sufficient for detecting small effects (f > 0.1) with 90% power at *α* = 0.05. All participants reported no previous history of neurological or psychiatric diagnosis and normal or corrected-to-normal vision. Using the revised version of Edinburgh Handedness Inventory (Milenkovic & Dragovic, 2013), the mean laterality quotient ([right-left]/[right+left] x100) was 99.5 (SD = 4.0). All individuals received 500 Kč (approx. €20) for their participation. The study was approved by the Ethics Board of the Institute of Psychology, Academy of Sciences of the Czech Republic. Written, informed consent was acquired from all of the individuals before their participation.

#### Materials

The experimental protocol consisted first of an emotional Go/No-go task, which is not reported here, and an SCR task that followed immediately afterwards. The SRC task involved the exact same stimuli, timing protocol and trial randomisation as described above, the only difference being that the task was administered in a fully within-subject manner; every individual responded to left and right stimulus hands presented at both counterclockwise and clockwise orientations. Each stimulus was presented separately in one of four blocks, the order of which was randomised to minimise practice effects. Blocks contained 30 trials of each condition: COM, INCOM, BASE, and catch (120 trials per block).

#### EEG Acquisition and Analysis

The SRC task was administered in E-prime 2.0.10.356 software (Psychology Software Tools; Pittsburgh, PA). The experiment was performed in dimly lit and sound-proofed room with electromagnetic shielding, with participants seated comfortably 160 cm from the presentation screen (visual angle = 58°). Stimuli were presented via a projector placed outside the room. Participants were instructed to minimise movement during the task. Continuous EEG was recorded using MR-compatible 256-channel EGI system GES400 with a sampling frequency of 1 kHz and Cz as the reference electrode. The maximum impedance was 50 Kohm. An EGI HydroCel 220 MR cap was used. Data were reduced to 204 electrodes by discarding channels placed on the face and neck, thereby minimising muscle artefacts. Pre-processing was conducted using a combination of routines from the EEGLAB toolbox and in-house solutions running under MATLAB 2017a. First, a bandpass Fast Fourier Transform filter of 1–40 Hz was applied. Independent component analysis was then performed to suppress ocular and cardiac artefacts. Electrode signals that recorded poorly (0-4%) were interpolated by spherical spline*.* Data were recalculated to an average reference and inspected visually for residual artefacts, which were discarded from further analyses.

For each condition, first we computed ERPs in sensor space. To be consistent with previous EEG investigations of the SRC task, we focused these analyses on temporal windows within which the N190 and P3 are expressed commonly: 170-220 and 310-430 ms post stimulus, respectively (Deschrijver et al., 2017b, 2017a; Rauchbauer et al., 2018). Trial segmentation included a 300 ms pre-stimulus baseline and 800 ms post-stimulus window. Clusters of electrodes expressing stable peak amplitudes in both compatible and incompatible conditions and across all stimulus hands were then identified (Supplementary Figure S3). Mean amplitudes pooled across these electrodes were calculated within each temporal window: for the N190, a left hemisphere (LH) cluster comprised CP1, P1, P3, 78, 89, 99, and 100; and a homologous right hemisphere (RH) cluster included CP2, P2, P4, 129, 130, 141, and 154. For the P3, mean amplitudes were pooled across P2, P4, P6, 141, 152, 154, and 163. Analyses were subsequently performed on these pooled mean amplitudes – namely, a 2 (*Condition*: COM, INCOM) x 2 (*Stimulus Hand*: LEFT, RIGHT) x 2 (*Orientation*: +90°, −90°) x 2 (*Hemisphere*: LH, RH) repeated-measures ANOVA on data from the N190 window, and a 2 (*Condition*: COM, INCOM) x 2 (*Stimulus Hand*: LEFT, RIGHT) x 2 (*Orientation*: +90°, −90°) repeated-measures ANOVA on data from the P3 window. Follow-up pairwise comparisons were performed with Bonferroni correction.

To estimate the intracerebral sources of scalp-recorded EEG, electrical source imaging was performed in Cartool (http://www.fbmlab.com/cartool-software), using the MNI head template and Locally Spherical Model with Anatomical Constraints forward model (Brunet, Murray, & Michel, 2011). An inverse solution was processed by the low resolution electromagnetic tomography (LORETA; Pascual-Marqui, Esslen, Kochi, & Lehmann, 2002). This functional imaging technique models the cortex as a 3D collection of discrete solution points (voxels) within MNI space, and by calculating current source density (CSDs) throughout this volume it provides an estimate of where in the brain the scalp-recorded EEG is generated. Although source accuracy is not perfect, the precision of source estimates from high-density scalp recordings has been shown to be around ±15 mm (Mégevand et al., 2014; Seeber et al., 2019). To identify brain regions in which neural signals are sensitive to orthogonal spatial compatibility, within the N190 and P3 windows separately we contrasted CSD maps between stimulus hands matched on orientation or anatomy but differing in their affordance of (complex) orthogonal-compatibility effects: LEFT_-90_ – RIGHT_-90,_ LEFT_-90_ – LEFT_+90_, RIGHT_+90_ – LEFT_+90_ , and RIGHT_+90_ – RIGHT_-90_.

To assess whether source estimates revealed the generators of scalp-recorded differences in AI between stimulus hands and orientations, we also computed ERPs in source space to explore evoked responses in parcellated brain areas—specifically, brain regions within which the scalp-recorded EEG was localised. Source space parcellation was performed with the Automated Anatomical Labelling (AAL) atlas (Tzourio-Mazoyer et al., 2002), and only centroid data from each brain region were kept. Proper dipole orientation in source space was specified by the refined dipole orientation approach (Coito, Michel, Van Mierlo, Vulliemoz, & Plomp, 2016).

### Results

#### Behaviour

Separate repeated-measures ANOVAs were performed on RT and accuracy data, with the within-subject factors *Condition* (COM, BASE, INCOM), *Stimulus Hand* (LEFT, RIGHT), and *Orientation* (−90°, +90°). Again, the results of analysis applied to accuracy data are presented in the Supplementary Materials (Figure S2). As before, differences among conditions are labelled according to the effects they represent (compatibility, facilitatory, or inhibitory).

#### *Response time*

As observed in Experiment 1, there were no main effects of *Stimulus Hand* (F[1, 68] = 0.83, *p* = 0.365, *r* = 0.11) or *Orientation* (F[1, 68] = 1.44, *p* = 0.234, *r* = 0.14). RTs did not differ statistically in response to left and right stimulus hands or between counterclockwise and clockwise rotations when collapsing across trial types. Furthermore, the main effect of *Condition* was again significant (F[2, 136] = 5.41, *p* = 0.005), with planned comparisons showing a significant compatibility (F[1, 68] = 4.49, *p* = 0.038, *r* = 0.25) and inhibitory effect (F[1, 68] = 9.49, *p* = 0.003, *r* = 0.35), but no facilitation (F[1, 68] = 1.42, *p* = 0.238, *r* = 0.14).

Follow-up contrasts of a significant *Condition*-by-*Stimulus Hand* interaction (F[2, 136] = 7.35, *p* = 0.001) demonstrated a compatibility effect in response to the left (13.58 ms, *p* < 0.001) but not the right stimulus hands (−2.36 ms, *p* > 0.999; F[1, 68] = 13.42, *p* < 0.001, *r* = 0.41), which is again consistent with Experiment 1. The inhibitory effect was not significant, however (F[1, 68] = 2.46, *p* = 0.121, *r* = 0.19). Although no facilitation was expressed either to the left (−1.81 ms, *p* > 0.999) or right stimulus hands (7.82 ms, *p* = 0.113), this effect was again significantly *reversed* for the latter (F[1, 68] = 5.30, *p* < 0.024, *r* = 0.27).

Similar to Experiment 1, a *Stimulus Hand*-by-*Orientation* interaction was observed only at trend level (F[1, 68] = 2.81, *p* = 0.098, *r* = 0.20). In contrast, the *Condition*-by-*Orientation* interaction was significant (F[1.82, 123.52] = 10.85, *p* < 0.001); planned contrasts showed that while a significant compatibility effect was present for the counterclockwise rotation (17.11 ms, *p* < 0.001), this was not true for the clockwise orientation (−5.89 ms, *p* = 0.301; F[1, 68] = 28.36, *p* < 0.001, *r* = 0.54), and a significant inhibitory effect was observed in response to the counter-clockwise (12.42 ms, *p* = 0.007) but not the clockwise stimulus rotation (4.80 ms, *p* = 0.727; F[1, 68] = 1.79, *p* = 0.186, *r* = 0.16). Furthermore, while no facilitation was observed for the counterclockwise rotation (−4.69 ms, *p* = 0.698), *a reversal* of this effect emerged again in response to the clockwise orientation (10.69 ms, *p* = 0.003; F[1, 68] = 9.57, *p* = 0.003, *r* = 0.35). In contrast to Experiment 1, however, and against our predictions, the three-way *Condition*-by-*Stimulus Hand*-by-*Orientation* interaction did not reach significance (F[1.99, 135.26] = 0.56, *p* = 0.570; Figure 1b).

#### *Inverted Efficiency*

**Fig. 2:**
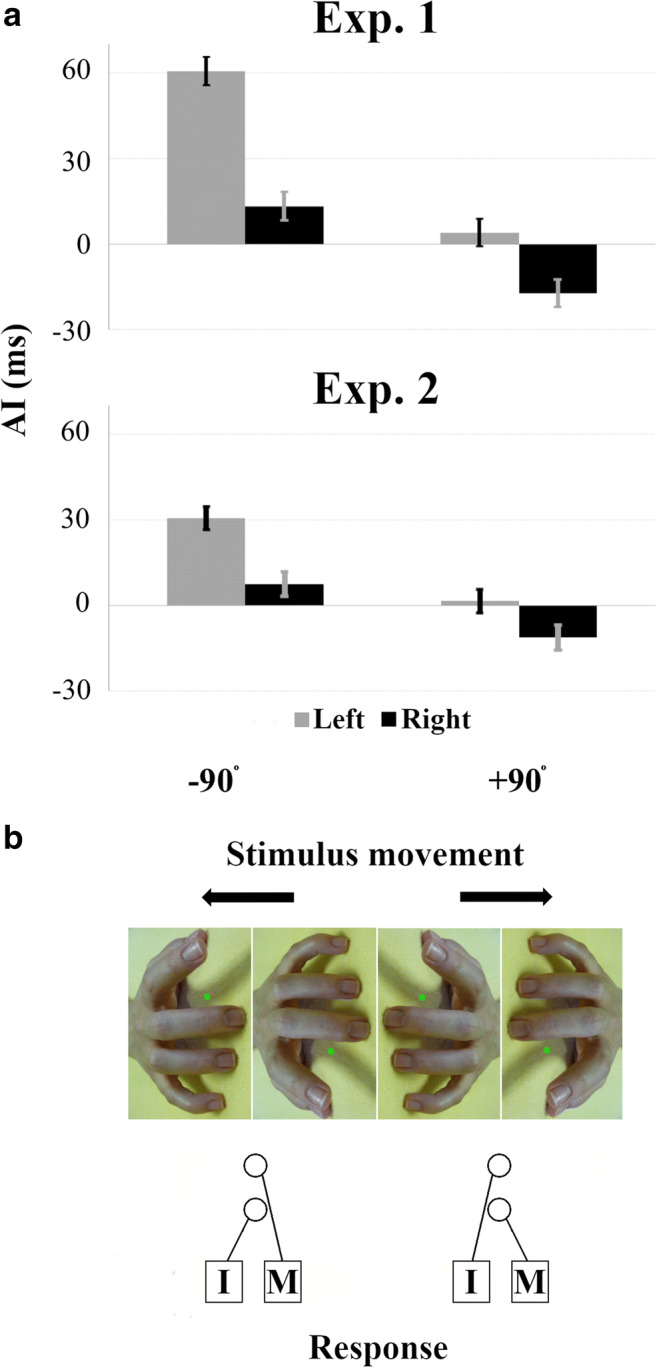
**a** Compatibility effects expressed by inverted efficiency scores (INCOM_IE_-COM_IE_) in response to left (*grey*) and right (*black*) stimulus hands on Experiment 1 (*top*) and 2 (*bottom*). **b** Illustration of complex orthogonal spatial compatibility for individual stimuli. Stimulus hands at counterclockwise (−90°; *left*) and clockwise rotations (+90°; *right*), all presenting imitatively compatible trials but differing in orthogonal spatial compatibility. I/M = index/middle finger response.

Analyses applied to IE scores showed significant main effects of both *Stimulus Hand* (F[1, 68] = 22.35, *p* < 0.001; *r* = 0.50) and *Orientation* (F[1, 68] = 36.03, *p* < 0.001, *r* = 0.59), with larger compatibility effects for the left (16.12 ms) compared with the right stimulus hands (−1.90 ms) and counterclockwise (19.09 ms) relative to clockwise rotation (−4.87 ms). While these data appear to converge with those from Experiment 1, suggesting that AI is maximal for the LEFT_-90_ stimulus and reversed for the RIGHT_+90_ stimulus, the *Stimulus Hand*-by-*Orientation* interaction was not significant (F[1, 68] = 1.55, *p* = 0.217, *r* = 0.15). Although this stands against our initial predictions, the *reversed* compatibility effect in response to RIGHT_+90_ was significant (*t*[68] = 2.59, *p* = 0.012, *d* = 0.62, 95% CI [−19.98, −2.59]; Figure 2a). This suggests that any imitative-compatibility effects elicited by this stimulus were overpowered by the strong influence of orthogonal up-left/down-right effects. Figure 2b illustrates this complex orthogonal relationship alongside these results.

#### Neurophysiology

#### *ERP data*

The 2 x 2 x 2 x 2 repeated-measures ANOVA applied to pooled mean amplitudes within the N190 window showed that none of the four main effects were significant (*p* ≥ 0.162). While the *Condition*-by-*Orientation* interaction failed to reach the threshold for significance (F[1, 65] = 3.335, *p* = 0.072, *r* = 0.22), it appeared to suggest a trend in the same direction as the behavioural data; specifically, stronger negativity on COM relative to INCOM trials for hand stimuli presented counter-clockwise (difference = 0.133 μV), but not those at a clockwise orientation (−0.025 μV). Similarly, follow-up contrasts of a significant *Condition*-by-*Stimulus Hand* interaction (F[1, 65] = 8.247, *p* = 0.006, *r* = 0.34) revealed that the compatibility effect was present in response to the left (0.189 μV, *p* = 0.011) but not right stimulus hands (−0.080 μV, *p* = 0.265). Although a significant *Stimulus Hand*-by-*Hemisphere* interaction emerged (F[1, 65] = 4.555, *p* = 0.037, *r* = 0.26), follow-up contrasts did not reveal any reliable effects (*p* ≥ 0.295). Lastly, a strong *Orientation*-by-*Hemisphere* interaction (F[1, 65] = 64.346, *p* < 0.001, *r* = 0.71) indicated that while the left hemisphere responded with stronger negativity to stimuli rotated clockwise (0.195 μV, *p* = 0.10), the right hemisphere showed this preferential response towards stimuli oriented counter-clockwise (−0.413 μV, *p* < 0.001). None of the other interactions were significant (*p* ≥ 0.176).

**Fig. 3:**
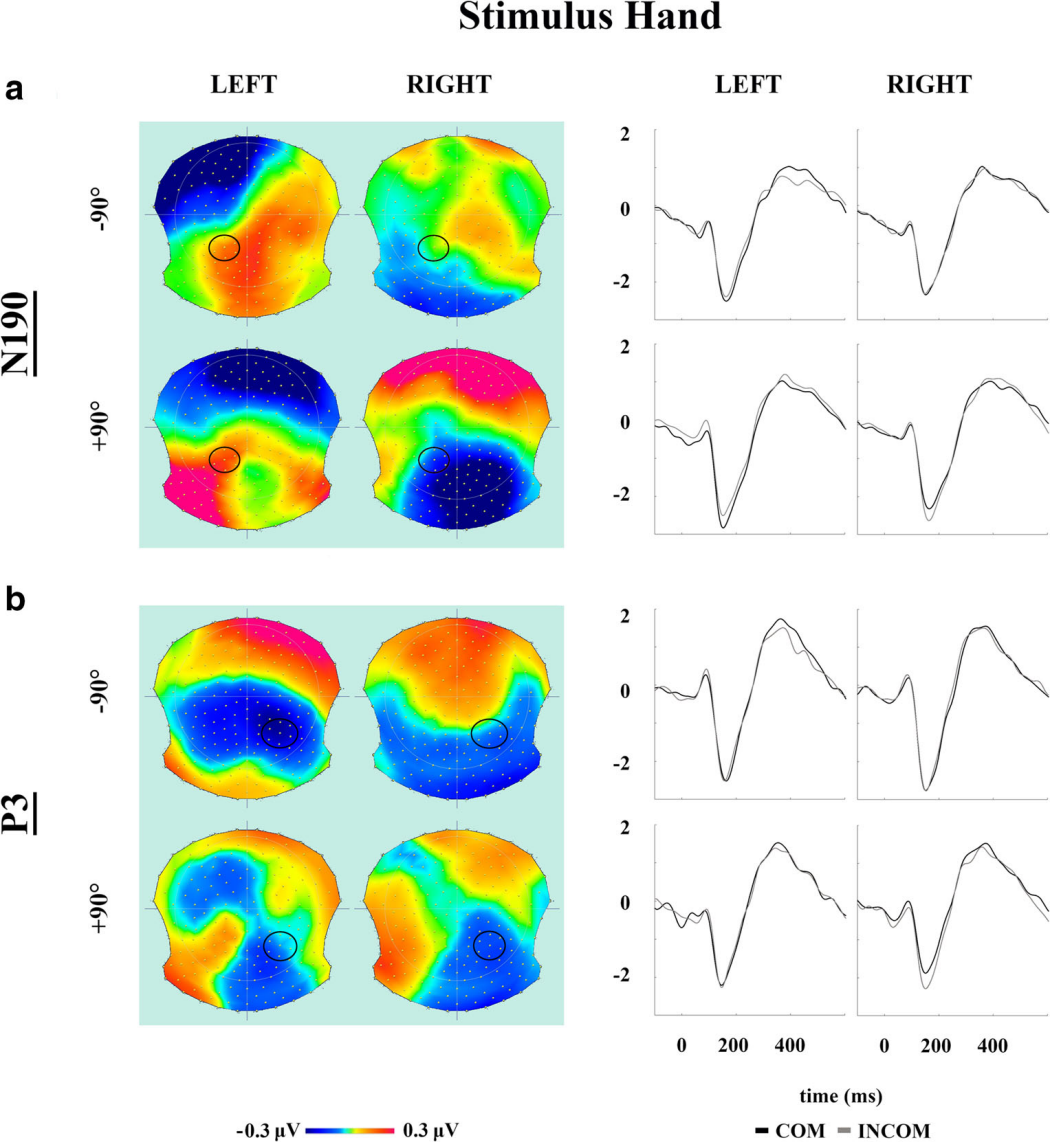
Differences in the compatibility effect expressed by event-related potentials (ERPs) for each stimulus hand. **a** Topographical maps of the N190 window (*left*) and ERP waveforms from the left hemisphere cluster of electrodes (*right*). **b** Topographical maps of the P3 window (*left*) and ERP waveforms. COM/INCOM = compatible/incompatible trials *(black*/*grey*)

The 2 x 2 x 2 ANOVA applied to pooled mean amplitudes from within the P3 window revealed a main effect of *Condition* (F[1, 65] = 10.819, *p* = 0.002, *r* = 0.38), with stronger positivity on COM relative to INCOM trials (0.137 μV). Interestingly, the main effect of *Orientation* also approached the threshold for significance (F[1, 65] = 3.716, *p* = 0.058, *r* = 0.23), suggesting that this later ERP component might be more responsive to actions performed by hand stimuli presented counter-clockwise compared with those rotated clockwise (0.119 μV). None of the other main effects or interactions were significant (*p* ≥ 0.117). Figure 3 presents topographies and pooled ERPs expressing the compatibility effect in both windows for individual stimuli.

#### *Brain-behaviour relationships*

**Fig. 4:**
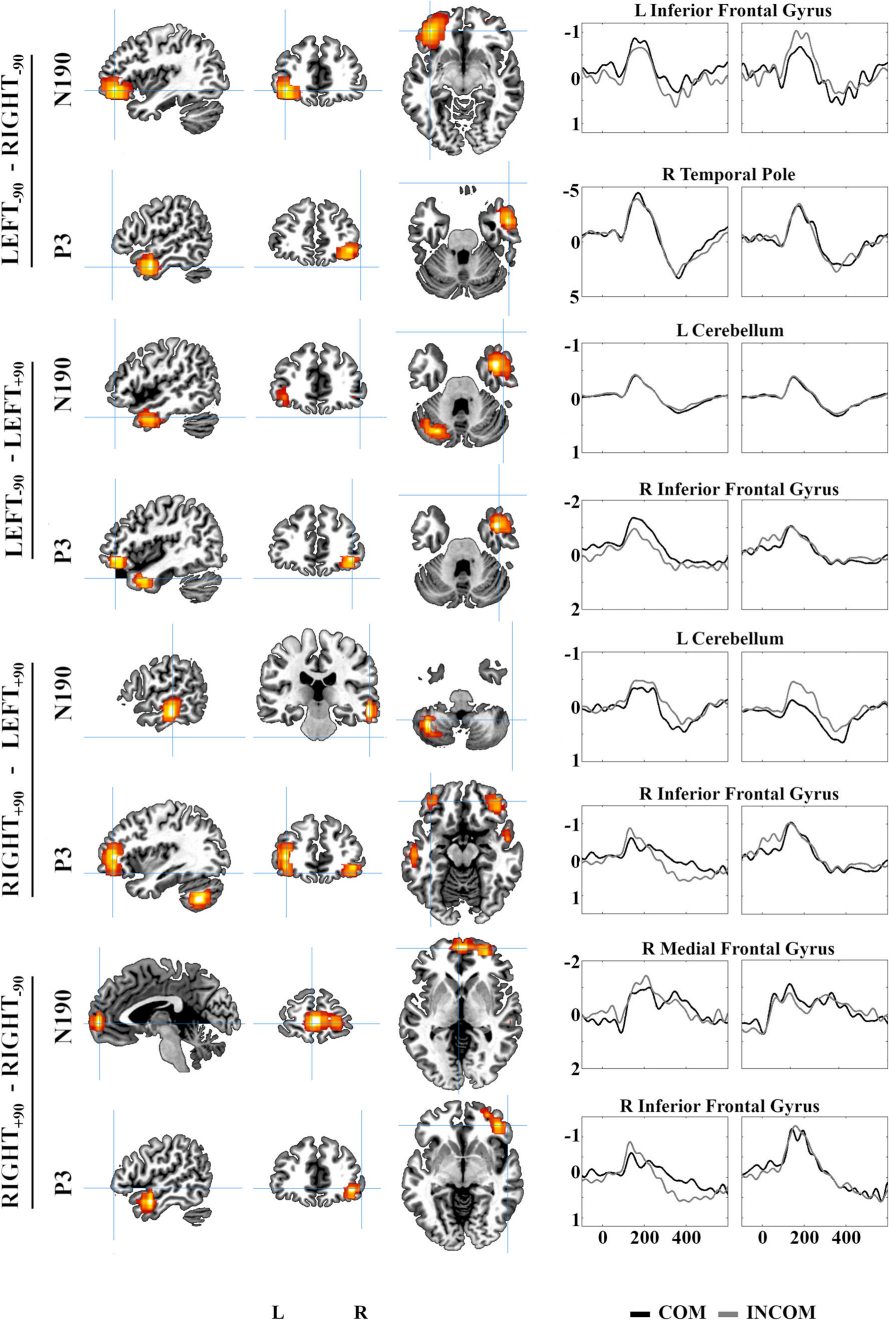
Differences in the compatibility effect between pairs of stimuli within the N190 and P3 windows. *Left*: Intracranial localisations, identifying maximal differences in current source densities (CSD; A/m2) between selected pairs of stimulus hands. *Right*: Source space event-related potentials (ssERPs), estimated within structures in which CSD differences were maximal between stimulus hands. For each comparison, the left and right graph present the ssERPs from the minuend and subtrahend stimulus, respectively. The y-axes present current source densities (10^-5^) and x-axes present post-stimulus times (ms). COM/INCOM = compatible/incompatible trials *(black*/*grey*).

To assess whether ERP indices of AI in response to the four stimulus hands were related to behavioural measures, for each participant we calculated a compatibility effect for both the N190 and P3 component that reflected their preferential response (COM-INCOM) and assessed their association with the behavioural (RT) index of AI (INCOM-COM) with Spearman correlations. These analyses revealed that larger compatibility effect in the N190 window acquired from the right hemisphere was associated negatively with behavioural expressions of AI in response to the RIGHT_-90_ stimulus hand (*r* = −0.351; *p* = 0.004; 95% confidence interval [CI] [−0.549, −0.125]; Figure 4). None of the other correlations were reliable (*p* ≥ 0.072; 95% CI [−0.480, 0.050]).

#### *Source Estimation*

**Fig. 5:**
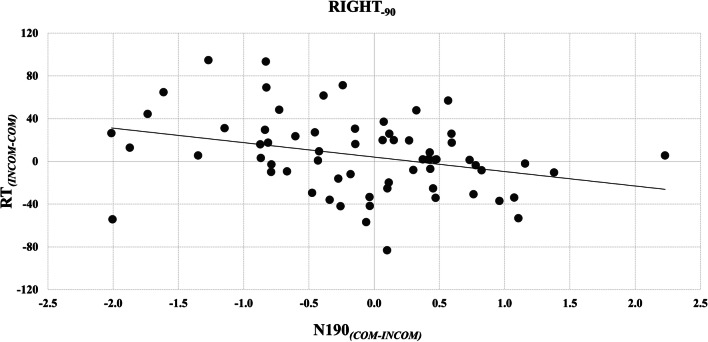
Brain-behaviour relationships. The behavioural index of AI (INCOM-COM) in response to the RIGHT_-90_ was associated negatively with the magnitude of compatibility effect (COM-INCOM) expressed by the N190 ERP component in the right hemisphere.

In the N190 window, contrasts between CSD maps identified a sensitivity to orthogonal compatibility after controlling for anatomical compatibility and orientation within the left IFG (LEFT_-90_ – RIGHT_-90_); right temporal pole and left cerebellum (LEFT_-90_ – LEFT_+90_); right middle temporal gyrus (MTG) and left cerebellum (RIGHT_+90_ – LEFT_+90_); and the right medial PFC (RIGHT_+90_ – RIGHT_-90_). For the P3 component, localisations were maximal in the right IFG and temporal pole (LEFT_-90_ – RIGHT_-90_); right IFG and temporal pole (LEFT_-90_ – LEFT_+90_); bilateral IFG and MTG, and the left cerebellum (RIGHT_+90_ – LEFT_+90_); and right IFG and MTG (RIGHT_+90_ – RIGHT_-90_; Figure 5). Source-space ERPs estimated within AAL-composite anatomical masks containing CSD peaks revealed similar patterns of change in both the magnitude and direction of compatibility effects that we observed in the scalp recordings. Most notably, both the N190 and P3 expressed a reversal between the LEFT_-90_ and RIGHT_-90_ stimulus within the left IFG, and between the RIGHT_+90_ and RIGHT_-90_ stimulus within the medial prefrontal cortex.

### Discussion

The behavioural results from Experiment 2 reveal a pattern of AI across stimuli mirroring that observed in the first experiment; a compatibility effect was elicited by left but not right stimulus hands, and only for the counterclockwise rotation. The exact same pattern was shown by the inhibitory effect, but there was a reversed facilitation for the right stimulus hand rotated clockwise – observing compatible actions performed by this right hand, which should facilitate action execution by evoking imitative tendencies, actually served to *interfere* with performance. This reversal provides further evidence that AI partly reflects overlearned orthogonal spatial associations that exert influences over behaviour strong enough to overpower any imitative effect resulting from the topographical similarity of observed and executed behaviour. In this light, AI measured on SRC tasks employing left stimulus hands rotated counterclockwise, which afford such confounding influences, is driven largely by nonsocial, or domain-general, spatial associations.

It is important to acknowledge that some of the interactive effects observed in the first experiment were not reproduced in the second, and the measures of AI are noticeably smaller than before. We suggest that this likely reflects practice effects. First, to manipulate stimulus orientation in a within-subject manner, it was necessary to administer four rather than two blocks of trials. Although any influence of practice should be minimised with the randomisation of block order, strong practice effects have been noted elsewhere; an up-right/down-left advantage has been shown to reverse after prior training with an opposite mapping using nonsocial stimuli (i.e., up-left/down-right; Iani et al., 2014; Proctor & Xiong, 2015). Nevertheless, the strong similarity in the pattern of AI across stimuli between Experiment 1 and 2 suggests that orthogonal-compatibility effects are fairly robust to these potential sources of practice.

The EEG data showed a pattern that resembled some of these behavioural findings. Within the N190 window, there appeared to be a trend towards greater ERPs in response to compatible relative to incompatible actions performed by counterclockwise but not clockwise-rotated stimuli. Furthermore, this direction of compatibility effect was expressed significantly in response for left but not right stimulus hands. Greater negativity for compatible compared with incompatible trials opposes the findings of previous research (Deschrijver et al., 2017b). In fact, upon closer inspection it appears that our experimental paradigm may have captured a different ERP component to the visual N190. Namely, the pattern of brain responses we have observed resemble more closely the N2 posterior-contralateral (N2pc)—a component characterised primarily by stronger negativity within posterior electrodes located contralateral to an evoking visual stimulus. This component showed greater negativity in the right hemisphere for stimulus hands rotated counterclockwise (left hemispace), but in the left hemisphere for clockwise-rotated stimuli (right hemispace). This might reflect fundamental differences in the rotated hand stimuli we have used in the present study and the non-rotated stimuli used elsewhere. Alternatively, it might reflect the superior spatial resolution achieved by our high-density EEG recording system, or our choice of recording parameters that permit a more detailed characterisation of electrocortical processes (e.g., the use of average rather than linked mastoid references; Hu, Yao, Bringas-Vega, Qin, & Valdes-Sosa, 2019; Yao et al., 2019). Regardless, because the N2pc component has been recorded on SRC tasks using nonsocial stimuli (Praamstra & Oostenveld, 2003; Valle-Inclán, 1996), we interpret this pattern of differential responding according to the spatial characteristics of rotated action stimuli as evidence that brain and behavioural responses on our SRC task partly reflect domain-general visuo-spatial processes rather than neurocognitive processes specific to imitation.

The general pattern of greater brain responses in the P3 window is consistent with previous studies: namely, greater positivity was observed in response to compatible relative to incompatible actions performed by all stimulus hands (Deschrijver et al., 2017a, 2017b; Rauchbauer et al., 2018). In contrast to the N190, stronger P3 responses also were observed in the right hemisphere for all stimuli. Together, this indicates that the later component was less sensitive to different combinations of hand anatomy and orientation. Furthermore, there was a strong trend toward greater P3 responses to stimulus hands at the counterclockwise relative to clockwise orientation. Given the corresponding pattern of behaviour in response to these stimuli, we speculate that the P3 component is more responsive to imitative- than confounding orthogonal-compatibility effects: AI was largest for the stimuli rotated counterclockwise, even after placing imitative and orthogonal effects in opposition (right stimulus hand), while any imitative effects afforded by clockwise-rotated stimuli appear to have been overpowered completely by complex orthogonal compatibility.

Interestingly, EEG data were associated with behaviour only when assessing responses within the N190 window to the right stimulus hand rotated counterclockwise: Greater early expressions of the compatibility effect (COM>INCOM), which appear to reflect a sensitivity to the spatial characteristics of stimuli rather than their topographical similarity with executed actions, were correlated *negatively* with behavioural indices of AI (INCOM>COM) in response to the right hand rotated counter-clockwise. Since this stimulus places imitative- and orthogonal-compatibility effects in opposition of one another, negative values of AI indicate that behaviour is driven more by orthogonal- than imitative-compatibility effects. This relationship might therefore suggest that early neurophysiological responses to orthogonal compatibility had the effect of reversing behavioural measures of AI—the observation of compatible and incompatible actions started to inhibit or facilitate action execution, respectively.

Source space analyses revealed a set of brain regions throughout prefrontal, anterior temporal and cerebellar cortices exhibiting compatibility effects that differed between stimulus hands matched on anatomy or orientation but varying in their affordance of orthogonal compatibility. The most consistent differentiations were localised around the IFG. Despite a considerable amount of research demonstrating the recruitment of IFG during imitative behaviour (Caspers, Zilles, Laird, & Eickhoff, 2010; Cross, Torrisi, Reynolds Losin, & Iacoboni, 2013; Molenberghs, Cunnington, & Mattingley, 2012), the extent to which this is driven by imitative compatibility between an executed and observed action—that is, their topographical similarity—remains unclear. Studies that have attempted to dissociate between imitative- and simple spatial-compatibility effects report either stronger IFG engagement during the imitation of spatially compatible hand actions (Koski et al., 2003) or identify the right IFG as a brain region engaged by both effects (Marsh et al., 2016). Importantly, IFG is associated with domain-general processes supporting inhibitory control (Duncan, 2010; Sebastian et al., 2013); the right IFG especially is believed to function as a “brake” to stop inappropriate actions completely or partially (Aron, Robbins, & Poldrack, 2014).

The sensitivity of the temporal pole/anterior temporal cortex (aTC) to orthogonal compatibility may seem surprising given the apparent lack of involvement from this brain region in previous SRC studies but makes sense when we consider its purported functional role and connectivity profile. The aTC is considered generally to serve as a hub node within a brain network involved in task-related semantic memory retrieval or “semantic control” (Ralph, Jefferies, Patterson, & Rogers, 2017). Via the uncinate fasciculus, the aTC is connected anatomically to both perirhinal cortex—a memory-related structure of the medial temporal lobe implicated in learning new associations (Ranganath & Ritchey, 2012)—and the IFG. Within this network, the aTC is believed to link sensory information with semantic representations under the control of IFG, permitting cognitive control over memory-guided behaviour (Jefferies, 2013). Interestingly, the response of aTC is dependent upon the difficulty of the task at hand (Barredo, Öztekin, & Badre, 2015); weaker associations appear to involve more effortful retrieval than stronger, more pre-potent ones. In this light, greater neural responses to stimuli affording orthogonal compatibility within cortical territories subsuming the IFG and aTC might reflect the semantic control needed to inhibit overlearned orthogonal spatial associations in favour of more transient, goal-directed stimulus-response mappings.

The left cerebellum also appeared to differentiate repeatedly between stimulus hands. While the cerebellum has received little direct research interest in the context of imitation, it is engaged consistently during social information processing (Van Overwalle, D’aes, & Mariën, 2015) and both action observation and execution (Molenberghs et al., 2012). Due to this response profile, the cerebellum has been suggested to support the mirror circuits of the brain; it is proposed to play a crucial role in understanding the actions of others, in the adaptive updating of predictions about oneʼs own and others’ actions, and even in self-other distinction (Caligiore, Pezzulo, Miall, & Baldassarre, 2013). In the present study, the differential engagement of the left cerebellum according to the presense of orthogonal spatial influences was observed primarily in the earlier time window, but also in the P3 when contrasting clockwise-rotated stimulus hands (LEFT_+90_ – RIGHT_+90_). We suggest that this might reflect more fundamental sensorimotor processes involved in motor-related conflict detection (Sokolov, Miall, & Ivry, 2017), rather than those specific to imitation *per se*. To understand precisely how orthogonal compatibility influences performance on SRC tasks with rotated hand stimuli, future studies should extend our preliminary findings and assess the relative influence of these confounding effects on motor-related ERP components. For example, other neuroscientific investigations have revealed that (in) compatibility between observed and executed actions influences cortical indices of movement preparation (readiness potential; Deschrijver et al., 2017a, 2017b) and initiation (premotor positivity; Rauchbauer et al. 2018).

## General Discussion

This study evaluated the stimuli used frequently in experimental investigations of the neurocognitive mechanisms behind imitative tendencies; specifically, we set out to characterise the confounding influence of orthogonal spatial-compatibility effects on automatic imitation (AI), at the behavioural and neurophysiological level. In two separate experiments, by manipulating systematically both hand anatomy and spatial orientation, AI was measured in response to four stimulus hands that differed in their affordance of orthogonal spatial-compatibility effects. The behavioural results provide strong and reproducible evidence that AI can be manipulated systematically (both increased and decreased, but also reversed completely) by orthogonal spatial influences, regardless of the topographical similarity between observed and executed actions. These behavioural measurements of AI were reflected partly in electrocortical responses; a sensitivity to orthogonal spatial compatibility was expressed in an early and, to a lesser degree, a later ERP component, which were localised to cortical regions encompassing bilateral IFG, aTC/temporal pole, and the left cerebellum. In the discussion that follows, we revise our previous interpretation concerning the direction of influence from orthogonal spatial compatibility and consider the cognitive and neurophysiological mechanisms that might drive this strong confounding influence.

Across two large-scale investigations, we observed the robust reversal of AI in response to a clockwise-rotated right stimulus hand. This was driven by a combination of reduced inhibitory and reversed facilitatory processes, revealing that the execution of finger actions was influenced by some factor other than the observation of topographically similar actions or (the inhibition of) imitative tendencies. The multiple asymmetric codes theory might go some way in providing an explanation for this finding (Cho & Proctor, 2002, 2003; Proctor & Cho, 2006); such complex orthogonal spatial-compatibility effects are proposed to result from the asymmetric coding of response and stimulus sets, which is determined by their relative spatial positioning. In fact, polarity correspondence is suggested to be a general principle of information processing and has been applied to the findings from various binary-choice tasks. This includes the Implicit Association Test, numerical parity judgement tasks, and word-picture verification tasks (Proctor & Xiong, 2015). If the asymmetric coding of left and right responses is driven by the influence of handedness on external space representations, which emerge through a lifetime of sensory-motor associations (Casasanto, 2009), polarity correspondence can also explain differences in the behaviour of left- and right-handed individuals on SRC tasks (Iani, Milanese, & Rubichi, 2014); an up-right/down-left advantage is reported when right handers map horizontal responses of their right hand onto a vertical stimulus display, but this reverses into an up-left/down-right advantage for left-handers responding with their left hand. Our interpretation of the shift in AI could be assessed directly in future research by investigating whether the pattern of AI observed in the present study is reversed in left-handed individuals.

Despite the strong opposing influences of orthogonal spatial compatibility on imitative effects, positive AI was observed in response to a right hand rotated counter-clockwise, suggesting that residual imitative effects remained for this stimulus. In contrast, the reversal of AI for a clockwise-rotated right stimulus hand implies that the weak (if any) imitative effects can be overpowered completely by orthogonal compatibility. This is further illustrated by responses to the left stimulus hands; while imitative- and orthogonal spatial-compatibility effects appear to have summed for the counterclockwise rotation, producing the largest AI, the positive but much smaller AI for the clockwise rotation suggests that it is driven by imitative compatibility alone. This difference in the relative contribution of imitative and orthogonal spatial effects elicited by different stimulus hand rotations has important methodological implications: Previous studies have attempted to estimate the dissociable influences of imitative and spatial compatibility by collapsing (averaging or summing) across left and right stimulus hands (Darda et al., 2018; Marsh et al., 2016). Our pattern of results indicates that this approach does not necessarily parse out the two components, however; each of our stimulus hands appeared to elicit different magnitude of overall AI, and the relative contribution of both influences is unknown. For this reason, we suggest that future experiments aiming to dissociate imitative and spatial compatibility estimate AI separately for each stimulus.

Turning now to our neurophysiological findings, source localisations of electrocortical responses reveal two brain areas exhibiting sensitivity to orthogonal spatial compatibility across all stimulus comparisons—one encompassing the IFG and another the aTC. The IFG has been implicated in action observation since the discovery of mirror neurons in a homologous area of the macaque brain (Rizzolatti & Craighero, 2004), and localised disruption of this brain region alters the pattern of AI observed in SRC procedures (Catmur, Walsh, & Heyes, 2009). The IFG also is implicated heavily in various aspects of response inhibition during nonsocial tasks; however, it is engaged during the selection of relevant and suppression of irrelevant stimuli on colour-naming Stroop tasks (interference resolution), withholding prepotent actions on Go-No-go tasks, overriding strong spatial stimulus-response associations on Simon tasks and cancelling initiated actions on Stop Signal paradigms (for a meta-analysis see Zhang, Geng, & Lee, 2017). While all of these processes involved in prioritising behaviour are conceivably necessary to control the involuntary tendency to imitate (Ramsey, 2018), response inhibition is a domain general neurocognitive mechanism unspecific to imitative tendencies. Indeed, the IFG is considered to be node of the (extended) multiple-demand network—a fronto-parietal brain system associated with a range of domain-general executive functions, such as inhibition, working memory, and the formation of task-dependent associations (Camilleri et al., 2018; Duncan, 2010).

The aTC/temporal pole also differentiated reliably between action stimuli affording both orthogonal and imitative compatibility from those isolating imitative effects from confounding spatial influences. As outlined above, the aTC is implicated heavily in semantic memory, the most convincing evidence coming from patients with semantic dementia whose progressive deterioration of semantic knowledge is correlated strongly with the degree of atrophy and hypometabolism in aTC (Visser, Jefferies, & Lambon Ralf, 2009). Together with the IFG, the aTC comprises part of a semantic control network associated with the retrieval of stored semantic representations in a controlled, goal-directed manner; more specifically, the retrieval of nondominant aspects of knowledge in the face of competing stronger associations to guide decisions (Jefferies, 2013; Lambon Ralph, Jefferies, Patterson, & Rogers, 2017). It is believed that the IFG serves to regulate the activation of semantic representations provided by aTC in a task-appropriate and context-sensitive fashion (Noonan, Jefferies, Visser, & Lambon Ralph, 2013). Interestingly, the strength of connectivity between these brain regions, at the level of both brain structure and function, is related to individual differences in semantic control (Wang et al., 2018).

We suggest that the sensitivity of the IFG and aTC to orthogonal spatial compatibility could reflect domain-general executive processes of cognitive and/or semantic control—processes necessary to override prepotent spatial associations in favour of weaker, task-relevant, stimulus-response mappings. Placing this within a theoretical framework of attentional control (Norman & Shallice, 2000), the orthogonal spatial dimension of stimulus hands activates schema representing well-learned input-output rules through neural systems involved in semantic processing, resulting in an automatic translation of the stimulus feature into a response code. When this competes with schema representing the goal-directed associations between imperative stimuli and finger movements, “contention scheduling” recruits a “supervisory attentional system” served by neural systems providing top-down inhibitory control to deactivate stronger associations in favour of weaker ones. Consistent with this interpretation, a meta-analysis of neuroimaging studies employing spatial inteference tasks (e.g., Simon task; Simon, 1969) reports that the inferior frontal junction, especially in the right hemisphere, appears to be involved in activating non-dominant but relevant stimulus-response mappings in the face of more automatic but irrelevant spatial associations (Cieslik et al., 2015). While both domain-general and imitation-specific processes might be recruited to control imitative tendencies (Ramsey, 2018), the use of stimuli that elicit behaviours driven largely, if not entirely, by orthogonal spatial associations makes it difficult to investigate the neurocognitive mechanisms involved specifically in imitative behaviour.

## Conclusions

This study presents robust evidence that the experimental stimuli employed frequently to investigate the neurocognitive mechanisms associated with imitative tendencies and their inhibition, might instead engage systems supporting unspecific spatial stimulus-response associations. Specifically, the strong influence of complex orthogonal spatial effects on behavioural and neurophysiological measures of automatic imitation elicited by these stimuli appears to reflect inherent biases associated with polarity correspondence. In this light, responses to these stimuli reveal less about specific mechanisms underpinning imitative behaviour and its suppression, and more about the domain-general principles of information processing. On the other hand, relatively large imitative effects were observed in response to a right stimulus hand rotated counter-clockwise, for which orthogonal spatial influences are controlled. This suggests that action observation can influence action execution—a phenomenon that polarity correspondence alone cannot explain. By demonstrating the strong confounding influence that spatial confounds exert on behavioural and neurophysiological indices of automatic imitation, the findings of this study should be used to guide future investigations into the neurocognitive mechanisms driving imitative tendencies.

## Supplementary Information

ESM 1 (DOCX 2042 kb)
