# Supplementary material for: Imitation or Polarity Correspondence? Behavioural and Neurophysiological Evidence for the Confounding Influence of Orthogonal Spatial Compatibility on Measures of Automatic Imitation
Source: Cogn Affect Behav Neurosci. 2021 Jan 12;21(1):212–30. doi: 10.3758/s13415-020-00860-y (PMC7994238; doi:10.3758/s13415-020-00860-y)
Supplement: Supplementary file 1 — (DOCX 2042 kb) [file 13415_2020_860_MOESM1_ESM.docx]

**SUPPLEMENTARY MATERIAL**

**Imitation or Polarity Correspondence? Behavioural and Neurophysiological Evidence for the Confounding Influence of Orthogonal Spatial Compatibility on Measures of Automatic Imitation.**

Kristína Czekóová, Daniel Joel Shaw, Martin Lamoš, Beáta Špiláková, Miguel Salazar, & Milan Brázdil


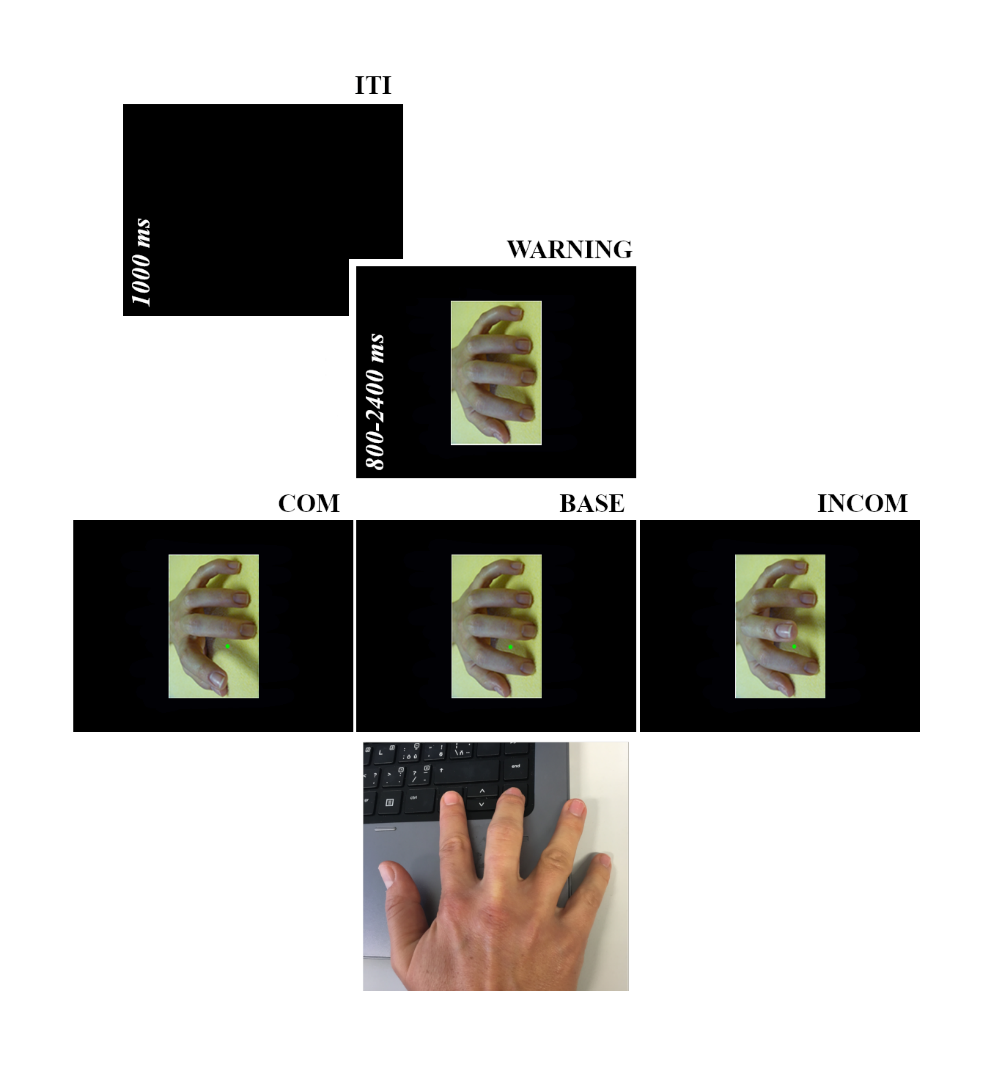


Supplementary Figure S1. Example trial sequence. An inter-trial-interval (ITI) was followed by a warning stimulus, after which an image depicting the end-point of a finger-lifting action was shown. Superimposed on this image was a coloured dot, which served as the imperative stimulus and defined the condition (Compatible [COM; *left*], Baseline [BASE; *middle*] or Incompatible [INCOM; *right*]).

**Experiment 1**

**Results**

Accuracy scores were assessed in a manner analogous to RT data: lower accuracy on INCOM compared with COM trials expresses a compatibility effect (AI), lower accuracy on INCOM compared with BASE trials reflects the inhibitory effect of observing an incompatible action and higher accuracy on COM relative to BASE trials indicates a facilitatory effect of observing an action that is compatible with the response signalled by the imperative stimulus. Where these differences appeared in the opposite direction (e.g., INCOM_ACC_ > COM_ACC_), we refer to these effects being *reversed*.

***Accuracy***

Consistent with the RT data, there were no significant main effects of *Stimulus Hand* (F[1, 290] = 1.34, *p* = .248, *r* = .07) or *Orientation* (F[1, 290] = 1.11, *p* = .294, *r* = .06), but a significant main effect of *Condition* (F[1.96, 567.68] = 65.37, *p* < .001); planned contrasts revealed a modest compatibility effect (F[1, 290] = 5.05, *p* = .025, *r* = .13), strong inhibition (F[1, 290] = 105.97, *p* < .001, *r* = .52) and a *reversed* facilitation effect (F[1, 290] = 85.27, *p* < .001, *r* = .48).

The *Condition*-by-*Stimulus Hand* interaction was also significant (F[1.92, 556.20] = 11.23, *p* < .001). Contrasts demonstrated that, as with RTs, when collapsing across rotations there was a compatibility effect for the left (-1.76 %, *p* < .001) but not the right stimulus hand (0.57 %, *p* = .488; F[1, 290] = 16.04, *p* < .001, *r* = .23). The inhibition effect did not reach significance (*p* = .482), and a *reversed* facilitation effect was smaller for the left (-1.24 %, *p* < .001) relative to the right stimulus hand (-3.22 %, *p* < .001; F[1, 290] = 15.70, *p* < .001, *r* = .23).

The *Stimulus Hand*-by-*Orientation* interactive effect was not significant (F[1, 290] = 0.05, *p* = .819, *r* = .01), but the *Condition*-by-*Orientation* interaction was (F[1.96, 567.68] = 12.36, *p* < .001). Follow-up contrasts of the latter revealed that, as with RT data, there was a significant Compatibility effect for the counter-clockwise (-1.88 %, *p* < .001) but not the clockwise rotation (0.69 %, *p* = .189; F[1, 290] = 23.51, *p* < .001, *r* = .27). Interestingly, a *reversed* facilitation effect was greater in response to the clockwise (-3.03 %, *p* < .001) relative to the counter-clockwise orientation (-1.43 %, *p* < .001; F[1, 290] = 10.97, *p* = .001, *r* = .19).

Finally, the three-way *Condition*-by-*Stimulus Hand*-by-*Orientation* was not significant (F[1.92, 556.20] = .04, *p* = .958). This goes against our primary predictions; while accuracy and RT data align in showing that AI was largest in response to the left relative to the right stimulus hand when combining across orientations, and present only for the counter-clockwise rotation when collapsing across anatomy, accuracy did not express greater AI for the LEFT_-90_ stimulus specifically.

**Experiment 2**

**Results**

***Accuracy***

In line with Experiment 1, the main effects of *Stimulus Hand* (F[1, 68] = 0.36, *p* = .549, *r* = .07) and *Orientation* (F[1, 68] = 0.52, *p* = .474, *r* = .09) were not significant for accuracy scores. We did, however, observe a main effect of *Condition* (F[2, 136] = 7.70, *p* = .001); when collapsing across stimulus hands and orientations, planned contrasts showed that a compatibility effect was not present (F[1, 68] = 1.01, *p* = .319, *r* = .12) but again there was significant inhibition (F[1, 68] = 12.84, *p* = .001, *r* = .40) and *reversed* facilitation (F[1, 68] = 9.55, *p* = .003, *r* = .35). There was no significant *Stimulus Hand*-by-*Orientation* (F[1, 68] = 0.274, *p* = .602, *r* = .06), *Condition*-by-*Orientation* (F[2, 136] = 0.14, *p* = .866), *Condition*-by-*Stimulus Hand* (F[2, 136] = 0.25, *p* = .783), or *Condition*-by-*Stimulus Hand*-by-*Orientation* interaction (F[2, 136] = 0.30, *p* = .743; see Figure 2B).


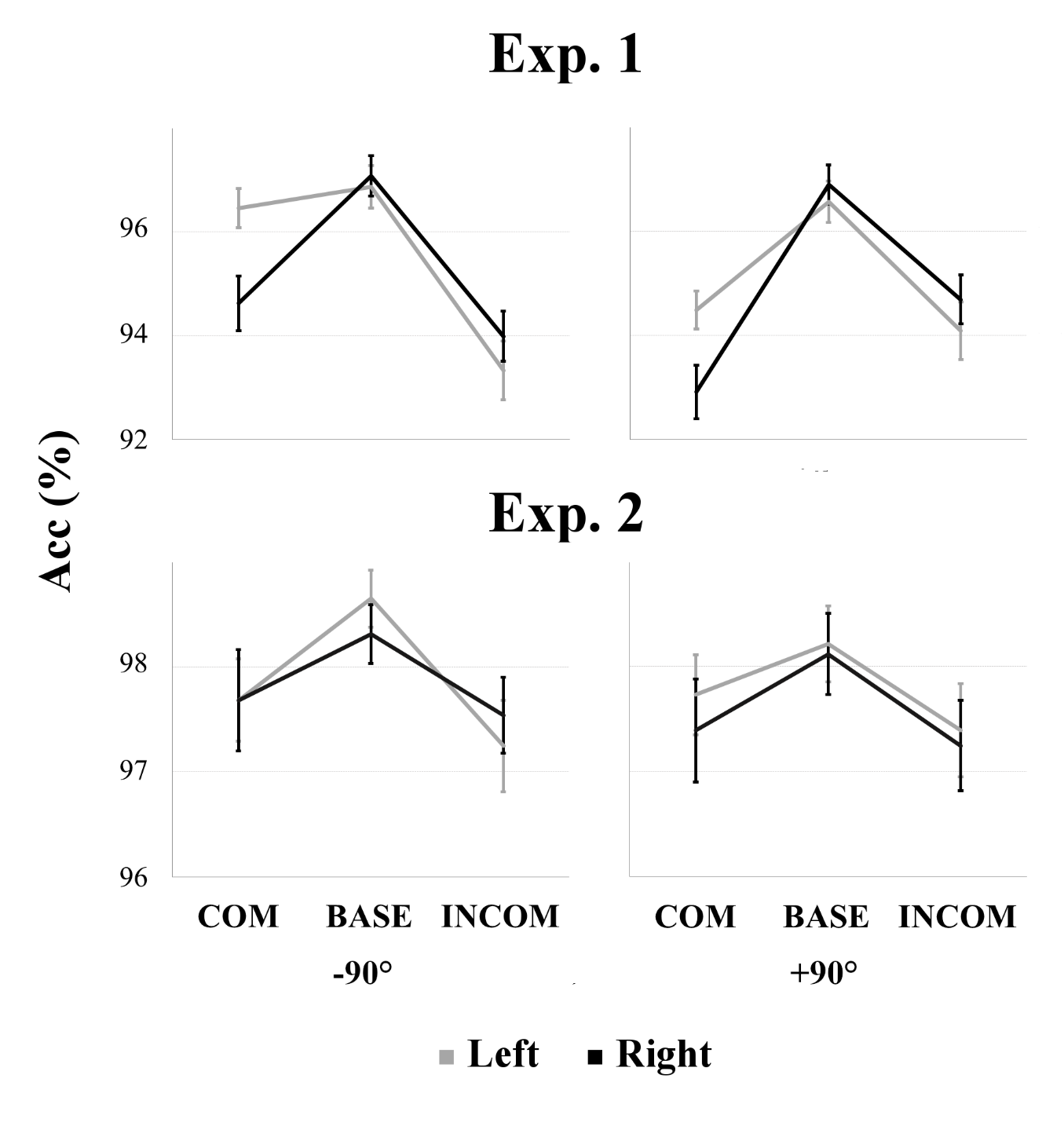


Supplementary Figure S2. Behavioural results: For Experiment 1 and 2, respectively, accuracies are presented for Compatible (COM), Baseline (BASE) and Incompatible (INCOM) trials in response to left (*grey*) and right (*black*) stimulus hands presented at a counter-clockwise (-90°; *left*) and clockwise orientation (+90°; *right*).


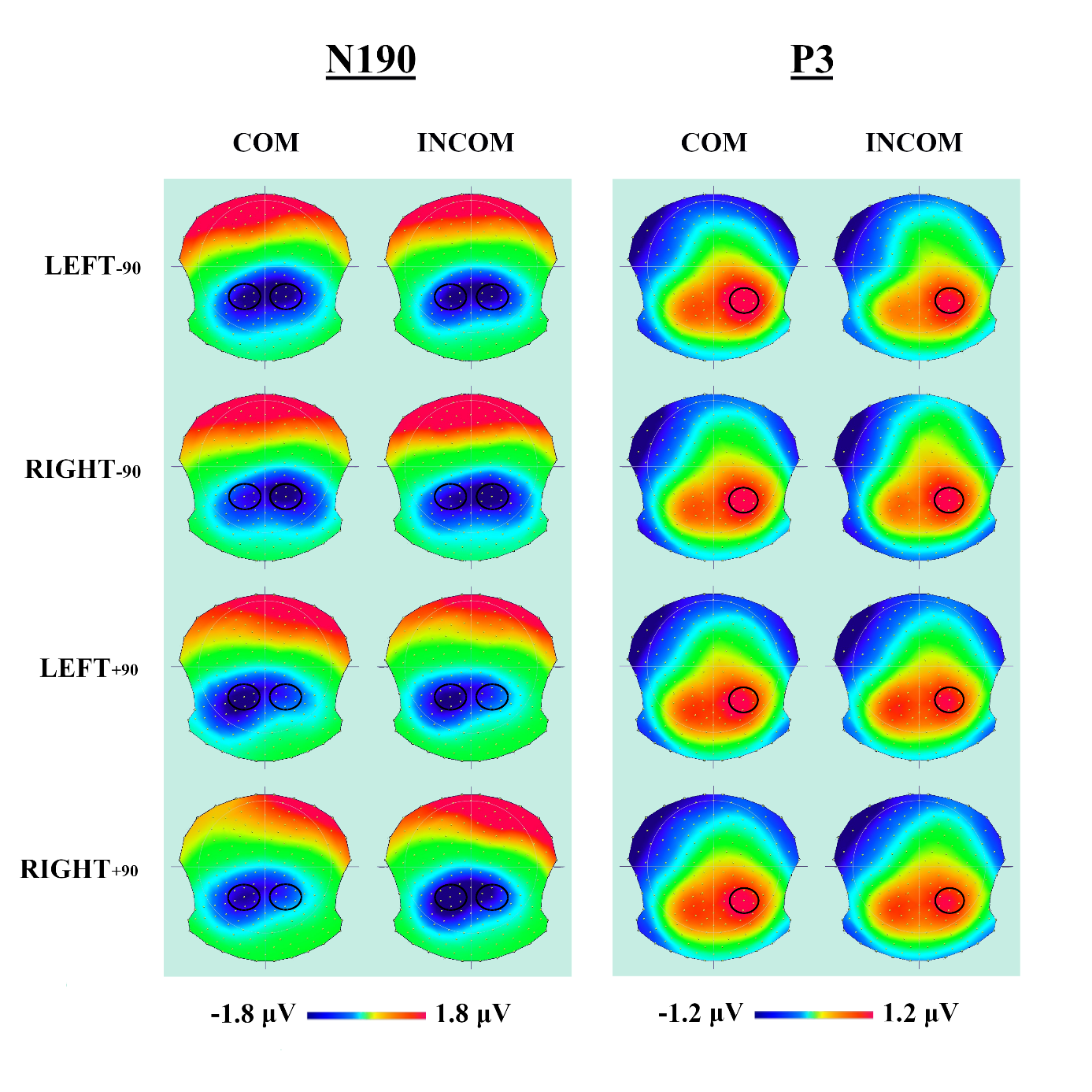


Supplementary Figure S3. Topographical maps illustrating the N190 (*left*) and P3 windows (*right*) for each stimulus hand and condition separately. Clusters of electrodes of interest are circled in black colour. Abbreviations: COM/INCOM = compatible/incompatible condition. *Top* to *bottom*: left and right stimulus hand in counter-clockwise (LEFT_-90_, RIGHT_-90_) and clockwise orientation (LEFT_+90_, RIGHT_+90_).
